# Enhanced membrane protein expression by engineering increased intracellular membrane production

**DOI:** 10.1186/1475-2859-12-122

**Published:** 2013-12-09

**Authors:** Mouna Guerfal, Katrien Claes, Oskar Knittelfelder, Riet De Rycke, Sepp D Kohlwein, Nico Callewaert

**Affiliations:** 1 Department for Molecular Biomedical Research, Unit for Medical Biotechnology, VIB, Technologiepark 927, 9052, Ghent, Belgium; 2 Department of Biochemistry and Microbiology, Laboratory for Protein Biochemistry and Biomolecular Engineering, Ghent University, K.L.-Ledeganckstraat 35, 9000, Ghent, Belgium; 3 Institute of Molecular Biosciences, University of Graz, Humboldtstrasse 50/II, A8010 Graz, Austria; 4 Department for Molecular Biomedical Research, Microscopy Core Facility, VIB, Technologiepark, 927, 9052 Ghent, Belgium; 5 Department of Biomedical Molecular Biology, Ghent University, Technologiepark, 927, 9052 Ghent, Belgium

## Abstract

**Background:**

Membrane protein research is frequently hampered by the low natural abundance of these proteins in cells and typically relies on recombinant gene expression. Different expression systems, like mammalian cells, insect cells, bacteria and yeast are being used, but very few research efforts have been directed towards specific host cell customization for enhanced expression of membrane proteins. Here we show that by increasing the intracellular membrane production by interfering with a key enzymatic step of lipid synthesis, enhanced expression of membrane proteins in yeast is achieved.

**Results:**

We engineered the oleotrophic yeast, *Yarrowia lipolytica*, by deleting the phosphatidic acid phosphatase, *PAH1*, which led to massive proliferation of endoplasmic reticulum (ER) membranes. For all eight tested representatives of different integral membrane protein families, we obtained enhanced protein accumulation levels and in some cases enhanced proteolytic integrity in the *∆pah1* strain. We analysed the adenosine A_2A_R G-protein coupled receptor case in more detail and found that concomitant induction of the unfolded protein response in the *∆pah1* strain enhanced the specific ligand binding activity of the receptor. These data indicate an improved quality control mechanism for membrane proteins accumulating in yeast cells with proliferated ER.

**Conclusions:**

We conclude that redirecting the metabolic flux of fatty acids away from triacylglycerol- and sterylester-storage towards membrane phospholipid synthesis by *PAH1* gene inactivation, provides a valuable approach to enhance eukaryotic membrane protein production. Complementary to this improvement in membrane protein quantity, UPR co-induction further enhances the quality of the membrane protein in terms of its proper folding and biological activity. Importantly, since these pathways are conserved in all eukaryotes, it will be of interest to investigate similar engineering approaches in other cell types of biotechnological interest, such as insect cells and mammalian cells.

## Background

Membrane proteins play critically important roles in a huge diversity of physiological processes. However, structural information on these proteins is scarce because of the multitude of experimental problems that need to be overcome to obtain a sufficient quantity of a detergent-solubilized, stable, homogeneous and monodisperse protein preparation from which well-ordered crystals for X-ray structural analysis can be grown. Only few membrane proteins have a natural abundance that is high enough to warrant purification from their native source, and heterologous overexpression is therefore required in most other cases. The expression systems that have been successfully used to produce membrane proteins for structure determination include mammalian cell lines, insect cells, bacterial cells and yeast. However, few expression systems have been specifically designed for the purpose of producing integral membrane proteins [1]. In part for that reason, only a small minority of membrane proteins can be readily overexpressed in a biologically active form, and this remains one of the bottlenecks on the way to a more robust workflow for membrane protein structure-function analysis. We here report on a eukaryotic endomembrane synthesis manipulation strategy for the overproduction of membrane proteins.

**Figure 1:**
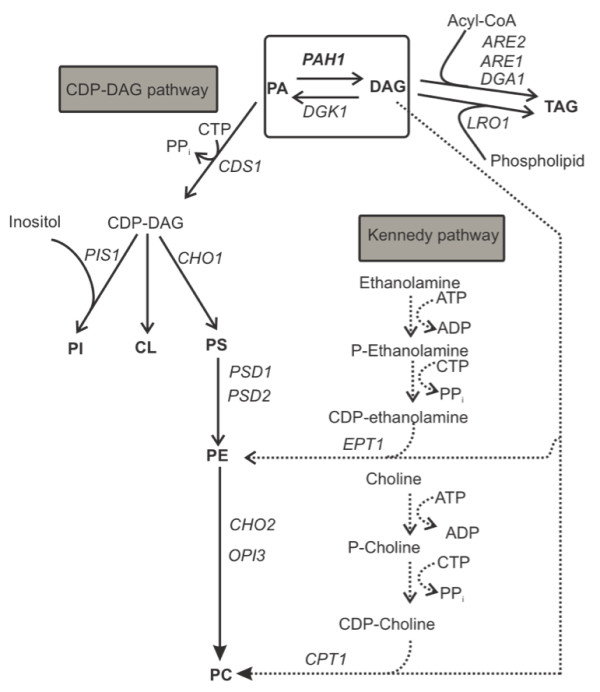
**Schematic presentation of the major steps in phospholipid biosynthesis in yeast [**[6]**].** Phosphatidic acid phosphatase, encoded by *PAH1,* catalyzes the dephosphorylation of PA to DAG, the precursor of the storage lipid TAG (there are 3 more genes coding for enzymes with this activity in yeast, but only *PAH1* is involved in the synthesis of TAG and the regulation of phospholipid synthesis [7]). All phospholipids can be derived from PA, with the exception of phosphatidylinositol which needs exogenous inositol input. PA, phosphatidic acid; DAG, diacylglycerol; TAG, triacylglycerol; PI, phosphatidylinositol; CL, cardiolipin; PS, phosphatidyl-serine; PE, phosphatidylethanolamine; PC, phosphatidylcholine.

As membrane proteins accumulate in the host cell’s intracellular and plasma membranes, we hypothesized that by providing a larger cellular membrane surface area the capacity to accommodate the overexpressed protein would increase. Many eukaryotic cells can absorb long-chain fatty acids (FA) and store them in cytoplasmic lipid droplets in the form of triacylglycerols (TAG) and steryl esters (SE) [2]. The uptake of fatty acids from the culture medium can also directly provide the precursors necessary for phospholipid synthesis and could facilitate the biogenesis of membranes, if their incorporation into TAG and SE lipid stores could be suppressed. Thus, our attention was drawn to *PAH1*, which encodes phosphatidic acid phosphatase Pah1p and which is a gate keeper for directing the flux of FA into TAG [3]. In *PAH1*-deleted *Saccharomyces cerevisiae*, phosphatidic acid (PA) cannot be converted to diacylglycerol (DAG) and, consequently, no TAG can be made via the Pah1 pathway (Figure 1). Informed by the massive ER/nuclear membrane proliferation seen in a *∆pah1* strain of *S. cerevisiae*[4] we investigated whether we could utilize this *∆pah1* phenotype to enhance membrane protein productivity. We chose to work with *Yarrowia lipolytica* because fatty acid-regulated promoters are well established for this organism [5] and we hypothesized that the ability to feed direct precursors for membrane lipids (i.e. fatty acids) would be most suitable to achieve enhanced membrane lipid biosynthesis.

As our previously reported data in *Pichia pastoris* indicated that co-induction of the unfolded protein response (UPR) can enhance membrane protein homogeneity and activity [8], we also evaluated the combined impact of endoplasmic reticulum (ER) membrane proliferation and UPR induction on the quantity and quality of the heterologously expressed membrane proteins.

## Results

### Identification and knockout of the *Yarrowia lipolytica PAH1* gene

The sequence of the *PAH1* gene of *Y. lipolytica* was identified through a homology search with the *Saccharomyces cerevisiae PAH1* gene (GeneID: 855201) against the genome of *Y. lipolytica*, using the Basic Local Alignment Search Tool (Blast) from NCBI. The Blast analysis identified the protein YALI0_D27016p, which shows some similarities to the nuclear elongation and deformation protein 1 of *Schizosaccharomyces pombe*, the S. *pombe* orthologue of Pah1p [9]. The sequence contains the conserved HAD-like domain with a DxDxT motif, in the middle of the protein sequence [10]. The family-characteristic N-terminal lipin domain was also identified (Additional file 1: Figure S1A).

A knockout construct for *PAH1* was made (Additional file 1: Figure S1B) to replace the *PAH1* coding sequence by the sequence of the *LEU2* selection marker [11]. The knockout was confirmed by PCR on genomic DNA (Additional file 1: Figure S1C).

### Impact of *PAH1* deletion on *Yarrowia lipolytica* growth

**Figure 2:**
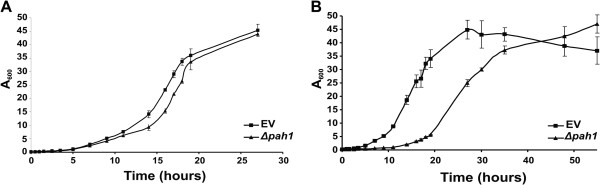
**Evaluation of the *Δpah1 *growth phenotype.** The growth phenotype of an empty vector strain and the *Δpah1* strain was compared on glucose and oleic acid as carbon sources. The empty vector strain (EV) is the PO1d *ΔOCH1* strain transformed with a plasmid containing the LEU selection marker cassette to rule out any effect of the selection pressure, compared to the *PAH1* knockout strain (*Δpah1*), which carries the LEU selection marker on the knockout construct. **A**. Growth on glucose as carbon source for 27 hours. Growth of the knockout was retarded only slightly and in the stationary phase, cells reached the same OD_600_ as the wild type strain. **B**. Growth on oleic acid as carbon source for 55 hours. Also here, the *PAH1* knockout strain has a prolonged lag phase but it reaches the stationary phase at the same OD_600_ as the empty vector strain.

As our purpose was to use the *Δpah1* strain for the large-scale production of membrane proteins, it was important to evaluate the impact of the *Δpah1* mutation on the growth characteristics of the strain on glucose and oleic acid, the two carbon sources used for protein production with *Y. lipolytica*. Growth curves for the wild type and the *Δpah1* strain were generated. Compared to the wild type control, growth of the mutant strain was only slightly retarded on glucose, and only during the early exponential phase (Figure 2A). Cells grown on oleic acid went through a longer lag phase but reached the stationary phase at the same optical density as the wild type strain (Figure 2B). These results show that the mutation does not appear to have a major impact on overall cellular physiology and does not present a serious obstacle to the utility of the strain for recombinant protein production.

### Membrane protein expression in the *Δpah1* and *Δpah1*/Hac1p strain

To begin to evaluate the impact of this lipid synthesis engineering step on the obtainable expression levels of heterologous integral membrane proteins, we chose to study the Adenosine A_2A_ receptor (A_2A_R) that belongs to the pharmacologically highly interesting family of G-protein coupled receptors (GPCRs). Radioligand binding assays are available to evaluate the functionality of the protein [12]. First, we generated a wild type *Y. lipolytica* strain expressing the A_2A_R from a single integrated gene copy under control of the oleic acid-inducible POX2 promoter. We then generated a *Δpah1* derivative of this strain, ensuring that the wild type and *Δpah1* strains expressed the A_2A_R in the same genetic background.

Furthermore, we studied whether this *Δpah1* membrane capacity engineering could be usefully integrated with a concomitant enhancement of the ER protein quality control capacity as afforded by induction of the unfolded protein response. The UPR in yeast is triggered by non-canonical splicing of the *HAC1* mRNA [13]. We identified the splice event in *Y. lipolytica* (as also identified since in [14]) and generated an expression construct for the spliced active *Yarrowia HAC1*, driven by the POX2 promoter. Subsequently, both the wild type and the *Δpah1* A_2A_R-expressing strains were transformed with this expression vector.

**Figure 3:**
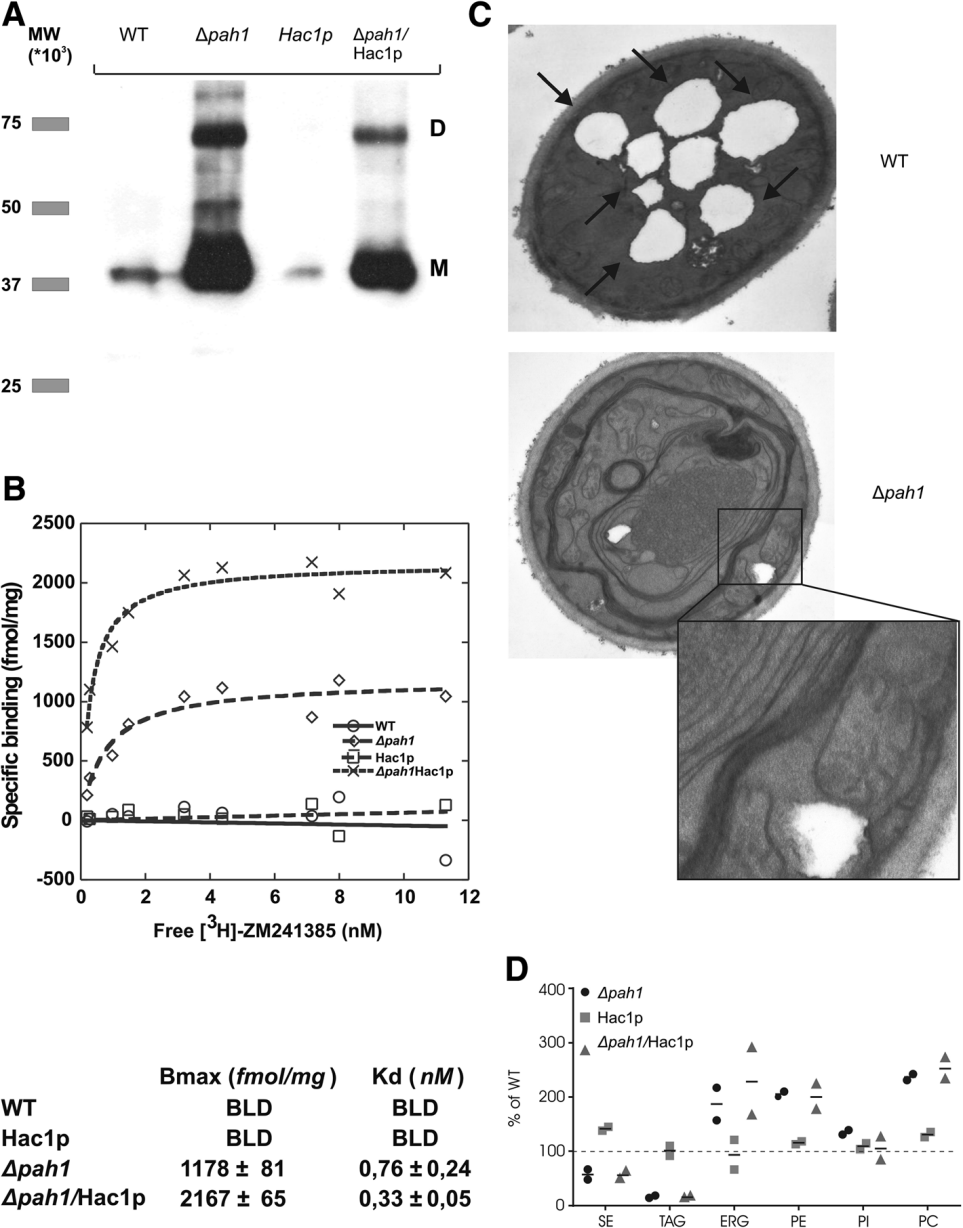
**Analysis of A**_**2A**_**R expression and membrane expansion in the *Δpah1 *strain. A**. Western blot analysis of A_2A_R in 10 μg of total membrane proteins of wild type, *Δpah1*, *HAC1* co-overexpression and *Δpah1*/Hac1p strains. **B**. Radioligand binding studies on membranes expressing the A_2A_R. Deletion of the *PAH1* gene leads to a strong increase (over 20-fold) in production of ligand-binding receptor. Artificial induction of the UPR reduces the quantity of receptor produced, but strongly enhances the radioligand binding activity, indicating enhanced receptor protein quality. **C**. EM pictures were taken after 48 hours growth on oleic acid of both wild type and *Δpah1* cells. When the knockout cells are grown on oleic acid, whirls and stacked layers of proliferated membrane become apparent. Also, few or no lipid droplets are present in the knockout strain, while big droplets are present in the wild type cells (arrows). **D**. HPLC-LSD analysis of the major cellular lipid classes derived from the strains grown on oleic acid. Lipid levels were quantified relative to those in the wild type parental strain (represented as 100%, broken horizontal line). The values derived from two independent experiments are shown, with their average represented by the short horizontal lines.

The expression levels of A_2A_R in the different strains were determined by western blot analysis (Figure 3A). The results demonstrate that the *Δpah1* mutation very strongly enhanced the expression levels of the GPCR. qPCR analysis showed that the mRNA levels for the GPCR were only slightly enhanced in the *Δpah1* strain (non-significant, P = 0.083, Mann-Whitney U-test, n = 8) (Additional file 2: Figure S2), indicating that stronger promoter activation and/or altered mRNA metabolism in the knockout is not the main causative factor for the strongly enhanced membrane protein accumulation. The *Δpah1*/Hac1p combination strain showed a somewhat lower production yield of the A_2A_ receptor than the *Δpah1* strain (Figure 3A). We next performed A_2A_R ligand binding studies on total membrane protein extracts to study whether the enhanced receptor expression levels also correlated with enhanced levels of ligand binding-competent receptor (Figure 3B). Under our test conditions, no binding of the ligand was detectable in the wild type POX2-A_2A_R strain, whereas in the derived *Δpah1* strain, B_max_ was 1178 ± 81 fmol per mg of total membrane protein. The lack of detectable ligand binding in the wild type strain was most likely due to the very low expression level of the receptor or its poor folding, or both.

Although the protein expression levels were similar in the *Δpah1* and *Δpah1*/Hac1p combination strains, we observed almost 2-fold increased specific radioligand binding activity of the receptor when Hac1p was co-overexpressed (2167 ± 65 fmol per mg of total membrane protein). We speculate that this is due to enhanced ER-chaperone mediated folding. In addition, enhanced ER associated degradation (ERAD) may aid in degrading molecules that are not folded in a ligand-binding competent state.

We also assessed intracellular membrane morphology in the wild type and *Δpah1* strains by electron microscopy (EM) of cells cultivated to high cell density (which is relevant for heterologous protein production) on oleic acid (Figure 3C). Wild type cells growing on oleic acid massively overproduce TAG, which is stored in cytosolic lipid droplets. In stark contrast, EM analysis of the *Δpah1* strain revealed massive intracellular membrane proliferation and almost complete absence of lipid droplets. This observation is consistent with the metabolic function of Pah1p to regulate the flux of phosphatidic acid into TAG synthesis.

The enhanced lipid fluxes towards membrane lipids in the *Δpah1 Y. lipolytica* strain grown on oleic acid were confirmed by HPLC-light scattering detection analysis of cellular lipid content and composition (Figure 3D): in the *Δpah1* mutant, the TAG pool was reduced approximately 5-fold, and the SE pool was reduced about 2-fold as compared to the wild type strain, whereas the levels of the membrane lipids ergosterol (ERG), phosphatidylethanolamine (PE) and phosphatidylcholine (PC) were all increased about 2-fold. The levels of phosphatidylinositol were similar to the wild type strain. *HAC1* overexpression in the *Δpah1* background did not alter this lipid composition, excluding the possibility that the *HAC1* overexpression effect on membrane protein quality was due to membrane lipid alterations (Figure 3D).

### The *Δpah1* mediated membrane protein overexpression is broadly applicable

**Figure 4:**
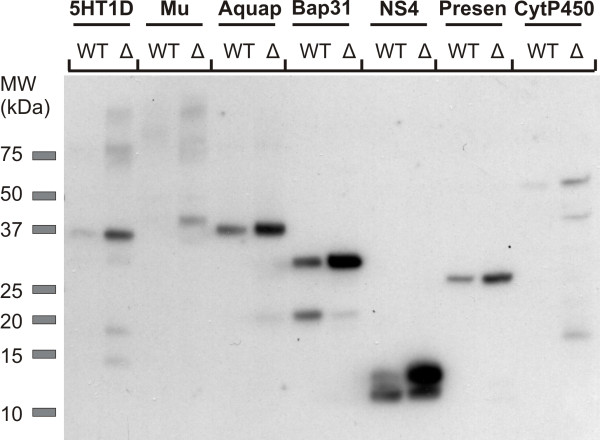
**Expression analysis of seven other membrane proteins in the wild type and *Δpah1 *strain.** Membrane protein expression, under control of the POX2 promoter for induction on oleic acid, was performed for 24 hours and 10 μg of total membrane proteins was analysed by western blot. For all seven membrane proteins tested, a moderate to strong enhancement of protein yield was observed. In some cases (Bap31 and NS4) there was also a reduced proteolysis of the protein. 5HT1D, 5-hydroxytryptamine 1D receptor; Mu, mu opioid receptor; Aquap, aquaporine Aqy1; Bap31, B-cell associated receptor 31; NS4, non-structural protein 4; Presen, presenilin; CytP450, cytochrome P450; WT, wild type; Δ, *Δpah1* strain.

To explore whether the massive intracellular membrane expansion seen in the *Δpah1* strain was specific to A_2A_R or had a beneficial effect on membrane protein yield and function in general, we studied expression of seven additional integral membrane proteins: the human 5HT_1D_ receptor, human mu opioid receptor, aquaporin Aqy1 of *Pichia pastoris*, human B-cell associated receptor 31, porcine respiratory virus (PRV) NS4, presenilin/SPP homologue of *Methanoculleus marisnigri JR1*, and human cytochrome P450 2D6. For all of these proteins, as for the A_2A_R described above, we first generated expressing clones and then knocked out *PAH1* in all of these strains. While significantly expanding the effort, this approach ascertains that differences in protein expression between the wild type and the mutants are not due to e.g. differences in transgene insertion site or copy number. All of the expressed membrane proteins in our analysis show a moderate to strong increase in the expression level in the *Δpah1* strain. In cases where proteolysis occurred in the wild type strain, this was much less the case in the *Δpah1* strains, for example for Bap31 and PRV NS4 (Figure 4). Thus, we conclude that deletion of the *PAH1* gene and enhanced membrane production in the presence of oleate provides a viable and widely applicable strategy for membrane protein expression.

## Discussion

In the last few years, several breakthroughs have been achieved in the ability to stabilize membrane proteins (especially GPCRs) by engineering the molecules themselves; these strategies have yielded several crystal structures of those membrane proteins that express relatively well in standard expression systems [15,16]. Together with these efforts, we expect that custom-engineered expression systems for integral membrane proteins, such as described here, will allow access to a much broader spectrum of membrane proteins than has been possible to date.

In the *Δpah1* strain, the cells are severely impaired in neutral lipid synthesis and consequently showed a strong reduction or absence of lipid droplets, which was also reported for *S. cerevisiae*[3]. The absence of this lipid droplet sink for fatty acids, makes the strain potentially susceptible to fatty-acid induced lipotoxicity. Indeed, the mutant strain showed a somewhat extended lag phase compared to wild type cells, however, it readily adapted to the presence of excess oleic acid and grew to similar cell densities as the wild type strain, in stationary phase. Although *S. cerevisiae* strains defective in neutral lipid synthesis (*Δare1 Δare2 Δdga1 Δlro1*) also show an extended lag phase on oleic acid and eventually reach wild type levels of cell density [17], this adaptation is likely due to second site suppressors [18]. Thus, *Yarrowia* – an oleaginous yeast – is much more tolerant to alterations in fatty acid fluxes. In any case, since a similar culture density is obtained with the *Yarrowia Δpah1* strain as with the wild type, this strain is entirely suitable for biotechnological protein production. All work reported here is based on simple shake flask cultivation with single-copy strains, thus, further strategies to enhance the yield of the strains developed in this study include optimal oleic acid feeding in controlled bio-fermentors, and exploring the effect of gene copy number increases. In addition, newer generations of stronger promoters for *Yarrowia* have recently been described [19,20] and will be useful to enhance the obtainable expression levels.

Importantly, the unfolded protein response and the master lipid flux regulatory role of Pah1p orthologues (lipins) [21], are conserved in all eukaryotes, including insect cells and mammalian cells. Technology to knock down or knock out genes in these organisms is now readily available [22,23]. Therefore, there is scope for applying the same membrane engineering approach to these other frequently used (membrane) protein expression hosts. However, careful exploration of an appropriate match between the carbon source and the promoter systems will be required for other biotech cell types in which the *Δpah1* manipulation is attempted and this will be the subject of further studies. For example, while *Pichia pastoris* is widely used for heterologous protein expression and extremely strong methanol-inducible promoters are available, our preliminary data (unpublished) suggest that C1-metabolism is not compatible with *Δpah1*-mediated membrane expansion: the membranes appear to be autophagocytosed, obliterating any beneficial effect on membrane protein expression yields. However, the rapidly expanding genetic toolbox for *Pichia*[24] will allow to match appropriate promoter systems with carbon source feeding strategies that allow for *Δpah1*-mediated stable membrane surface expansion.

## Conclusions

We conclude that *Δpah1*-mediated redirection of lipid synthesis fluxes away from storage lipids and towards membrane lipids, leads to a strong intracellular membrane proliferation especially when the cells are fed with fatty acids. This membrane expansion affords enhanced expression levels and proteolytic integrity of integral membrane proteins. We furthermore conclude that co-induction of the UPR in such *Δpah1* strains improves the quality of the overproduced membrane proteins. The engineered pathways are conserved in all eukaryotes, which offers a scope for application in other yeasts, insect cells and mammalian cells. To our knowledge, this is the first successful attempt to engineer the membrane synthesis machinery of a eukaryotic expression system to customize cells for accommodating integral membrane proteins. The improved yield, stability and activity of membrane proteins in these mutant strains are likely to enhance progress on their functional and structural characterization.

## Methods

### Strains

*E. coli* MC1061 was used for standard molecular biology manipulations. *E. coli* was grown in LB medium supplemented with the appropriate selection antibiotics. *Yarrowia lipolytica* PO1d *∆OCH1* was used for analysis and protein expression. This strain is a knockout for the *OCH1* mannosyltransferase in the wild type PO1d strain (CLIB 139), which results in the absence of N-glycan hypermannosylation, which is beneficial for more homogenous glycoprotein production [25].

### Transformation

Transformation was performed by the lithium acetate method as described by Barth and Gaillardin [26].

### Generation and identification of a *PAH1* knockout strain

A *PAH1* knockout strain was generated according to the protocol described by Fickers *et al*. [11] and as depicted in Additional file 1: Figure S1B. Genomic DNA was isolated using the MasterPureTM Yeast DNA Purification Kit according to the manufacturer’s instructions (Epicentre Biotechnologies, WI, USA). Clones that were prototrophic for leucine were then genotyped by PCR with primers pah1ylPfw07-010 (5′-GCGGCCGCGAAGACGGTGAGTATGGCCATC-3′) and pah1ylTrv07-007 (5′-GCGGCCGCCCAAACCATGCATACAAATCAG- 3′. PCR was performed with Phusion polymerase (Thermo Scientific, MA, USA): 5 min at 98°C, followed by 25 cycles of 1 min at 98°C, 20 sec at 60°C, and 45 sec at 72°C. A band of 3268 bp was expected for the wild type *PAH1* and a band of 2966 bp for the *Δpah1* strain. Random integration would lead to the presence of both bands.

### Cloning of spliced form of *Y. lipolytica HAC1* and construction of Hac1p expression plasmid

The spliced form of *HAC1* mRNA from *Y. lipolytica* PO1d was isolated as described earlier [8]. In brief, UPR was induced by adding DTT to 5 mM to an exponentially growing culture. RNA was isolated and reverse transcribed as described. The spliced cDNA was cloned into the *BamH*I/*Avr*II sites in plasmid JMP62hyg, which is a *Yarrowia* expression vector containing the POX2 promoter and a hygromycin resistance cassette. The resulting plasmid is called JMP62hygHAC1(s). The plasmid was cut with *Kpn*I before transformation to allow integration in the *POX2* promoter locus.

### Construction of the membrane protein expression vectors

The coding sequence of the Adenosine A_2A_ receptor was isolated from a fetal brain cDNA library with primers 5′-GAATGCAATGCCCATCATGGGCTCC-3′ and 5′-CCTAGGTCAAGCTGGAGCAACTTGAGAAG-3′, which include the *Bsm*I and *Avr*II restriction sites. The isolated gene was cloned in the pCR®-Blunt II-TOPO vector (Invitrogen). The insert in the resulting vector pTOPOA2A was sequence-verified. The open reading frame was then cloned in the vector pYLPUXdl2prepro, which contains a ura3d1 selection marker, the POX2 promoter and the secretion signal of the LIP2 gene for fusion to the coding sequence of the protein of interest. To obtain the construct expressing the A_2A_ receptor from the POX2 promoter, this vector was cut with *Fse*I and *Avr*II. The coding sequence of the A_2A_ receptor was isolated from the pTOPOA2A vector by *Bsm*I/*Avr*II digestion. The appropriate fragments were ligated and the final plasmid was called pPOX2-A2A. The genes of the seven other membrane proteins, fused to the LIP2 secretion signal, were produced using synthetic DNA technology (GenScript, NJ, USA) with terminal BamHI/AvrII restriction sites for cloning into the pYLPUXdl2prepro backbone.

### Growth curve

To analyse the growth phenotype of the *PAH1* knockout strain, a comparison was made with an empty vector (EV) strain. This EV strain is the PO1d *ΔOCH1* strain transformed with a NotI-linearized plasmid, pJMP62LEU, containing the LEU selection marker cassette, making this strain leucine-prototrophic, as the knockout strain. Cultures were grown overnight to saturation in YPD (1% yeast extract, 2% peptone, 2% dextrose) or YTO (50 mM phosphate buffer pH 6.8, 1% yeast extract, 2% tryptone, 2% oleic acid) and diluted the next day to an OD_600_ of 0.2. Incubation was continued and OD_600_ was measured periodically. For the cultures grown on medium containing oleic acid, the cells were spun down for 10 minutes at 13,000 rpm and washed once with 0.1N NaOH to avoid interference of oleic acid in the OD_600_ measurement. Samples were measured in triplicate.

### Small scale expression

Induction experiments for the Adenosine A_2A_ receptor were performed in 12.5 ml of culture medium in a 125-ml baffled flask, for the other membrane proteins, induction was performed in 2 ml of culture medium in 24 deep well plates. For all experiments, an overnight pre-culture was used to inoculate medium to an OD of 0.1. Cells were grown for 24 hours in YTD (1% yeast extract, 2% tryptone, 2% dextrose), washed once with water, and then resuspended in induction medium (50 mM phosphate buffer pH 6.8, 1% yeast extract, 2% tryptone, 2% oleic acid). Cells were cultivated for another 24 hours before harvesting.

### Membrane protein preparation

Cells (approximately 12 × 10^8^ cells) were pelleted by centrifugation at 1,500 × g and the pellet was resuspended in ice-cold disruption buffer (50 mM sodium phosphate buffer pH 7.4 supplemented with Complete protease inhibitor cocktail (Roche)). Cells were then broken by vigorous vortexing with glass beads in a mixer mill for 5 x 2 min at 4°C. Cell debris was separated from the membrane suspension by low speed centrifugation (1,000 × g, 30 min, 4°C). Total cellular membranous organelles were pelleted at 100,000 × g at 4°C for 60 min. For all membrane proteins analysed in Figure 4, the ultracentrifugation step was substituted for centrifugation at 13,000 × g at 4°C for 60 min, as this was found not to be required to enable western blot analysis, and to yield very similar results as the protocol involving ultracentrifugation. The membranous pellet was resuspended in 50 mM sodium phosphate buffer pH 7.4 supplemented with Complete protease inhibitor cocktail and snap-frozen in liquid nitrogen until further testing. The protein concentration of the membrane preparation was determined using the BCA reagent (Pierce, Rockford, IL) with BSA as a standard. Ten micrograms of total membrane protein were analyzed by western blot. The blot was blocked overnight in 0.05% Tween-20 and 3% casein in 1x PBS and probed with a 1/500 diluted primary mouse anti-Rho1D4 antibody, followed by a 1/3000 diluted secondary anti-mouse IgG peroxidase from sheep (Sigma Cat. n° NA931V). Protein bands were visualized with Renaissance western blot Chemiluminescence Reagent Plus (PerkinElmer).

### Ligand binding of Adenosine A_2A_ receptor

The procedures for studying binding to recombinant A_2A_ receptors have been described [12]. Briefly, 10 μg of total membrane proteins was incubated with different concentrations of the A_2A_R antagonist, [3H]ZM241385 (0.05-12 nM), in 500 μl of binding buffer (20 mM HEPES pH 7.4, 100 mM NaCl). Adenosine deaminase (0.1 U) was added to degrade the adenosine released from the membranes, and the membranes were incubated at 22°C for 1 hour. Non-specific binding was determined in the presence of 10 mM theophylline. Measurements were performed in duplicate. After incubation, bound and free ligands were separated on Whatmann GF/B filters pretreated with 0.1% polyethylenimine using a Brandel cell harvester. The filters were washed three times with binding buffer and the amount of bound radioligand was measured on a liquid scintillation counter. Kd and Bmax were determined by rectangular hyperbole curve fitting using KaleidaGraph software (Synergy Software).

### Electron microscopy

Samples were prepared for EM according to Baharaeen *et al*. [27]. Yeast cells were fixed for 2 h on ice in 1.5% paraformaldehyde and 3% glutaraldehyde in 0.05 M sodium cacodylate buffer, pH 7.2. After washing three times for 20 min in buffer, cells were treated with a 6% aqueous solution of potassium permanganate for 1 h at room temperature, to enhance membrane contrast. After washing three times for 20 min in buffer, cells were dehydrated through a graded ethanol series, including bulk staining with 2% uranyl acetate at the 50% ethanol step, followed by embedding in Spurr’s resin. Ultrathin sections of a gold interference colour were cut using an ultra microtome (Ultracut E; Reichert-Jung), post-stained with uranyl acetate and lead citrate in a Leica ultrastainer, and then collected on formvar-coated copper slot grids. They were viewed with a transmission electron microscope 1010 (JEOL, Tokyo, Japan).

### Reverse transcription quantitative PCR

Eight biological replicates of both the wild type strain and the *PAH1* deletion strain were grown and induced as described above. After 24 hours of induction, 6.10E8 cells were pelleted and washed twice with 0.1M NaOH at room temperature to completely remove oleic acid. Total RNA was prepared using the RiboPure yeast kit (Ambion, Life Technologies) according to the manufacturer’s protocol. 10 μg of total RNA was submitted to DNase treatment using the TURBO DNA-free kit (Ambion, Life Technologies). RNA integrity was confirmed for all sixteen samples using the Agilent RNA 6000 Pico Kit on the BioAnalyzer 2100 (Agilent Technologies). cDNA was prepared from 2 μg DNase-treated RNA using the iScript Synthesis Kit (BioRad) and a control reaction lacking reverse transcriptase was included for each sample. The RT-PCR program was as follows: 10 minutes at 25°C, 30 minutes at 42°C, 5 minutes at 85°C, and then cooling down to 12°C.

Real time quantitative PCR was done on a LightCycler 480 (Roche Diagnostics) using the SensiFast SYBR-NoRox kit (BioLine), in triplicate for each RNA sample, on a 384-multiwell plate, with 5% cDNA in a total volume of 10 μL. Primers were used at a final concentration of 10 μM. All primer pairs were generated using PrimerBlast (http://www.ncbi.nlm.nih.gov/tools/primer-blast) and verified for cross-specificity within the *Yarrowia* genome, as well as optimized for reduced secondary structure formation and reduced duplex formation. Primers used in the final experiment can be found in Additional file 3: Table S1. The following programme for cDNA synthesis was used: 3′ at 95°C, and 45 times 3″ at 95°C - 30″ at 60°C - 1″ at 72°C. The necessary controls (no reverse transcriptase controls, no template controls) were included. The stability of 10 candidate reference genes for normalization was analyzed in a pilot experiment (data not shown) using the genormPLUS algorithm, as implemented in the qbasePLUS software [28]. Based on these results, all gene expression values were normalized using the geometric mean of the genes PGK (Gene ID: 2910137) and QCR9 (Gene ID: 2906637). Determination of amplification efficiencies and conversion of raw Cq values to normalized relative quantities (NRQ) were performed using the the qbasePLUS software [28]. Statistical analyses of NRQs were done with the Prism 6 software package using the Mann-Whitney U-test.

### Lipid extraction

Lipid extraction of wild type, *PAH1* knockout, Hac1p overexpressing and *Δpah1/*Hac1p strains was performed. After cultivation as described above, cells corresponding to 20 OD_600_ units were harvested and disrupted with glass beads in chloroform/methanol 2:1 (v/v), by shaking in a Heidolph Multi Reax test tube shaker (Schwabach, Germany). Lipids were extracted according to Folch *et al.*[29].

### HPLC-LSD

The chromatographic setup consisted of an Agilent 1100 combining pump, injector, precooled sample manager (4°C) and column oven (40°C). For detection of the lipids, a Sedex 85 evaporative light scattering detector (Sedere, France) was used. Data acquisition was performed by the Chemstation software (B 04.01). A ternary gradient (modified from [7]) with a Betasil Diol column (100 × 4.6 mm, particle size 5 μm, Thermo) was used to separate the various lipid classes. Neutral lipid standards were purchased from Larodan, except for ergosterol (Acros Organics) and cholesterylpalmitate (Sigma Aldrich). Neutral lipid and phospholipid (Avanti Polar Lipids) standards were prepared as 1 mg/ml stock solutions in chloroform/methanol 2:1 (v/v). Calibration curves were measured from 2.7 μg/ml to 350 μg/ml. The injection volume for all calibration standards and samples was 10 μl.

## Competing interests

The authors declare to have no competing financial interests.

## Authors’ contributions

MG and KC designed and performed experiments, analyzed and interpreted data and drafted the manuscript. RDR performed the EM analysis, OK conducted the HPLC-LSD measurements and interpreted the results, SDK supervised and interpreted the lipid analysis experiments. N.C. initiated and designed the study, coordinated the project, interpreted data and co-wrote the manuscript. All authors read and approved the final version of the manuscript.

## Supplementary Material

Additional file 1: Figure S1 *Yarrowia lipolytica PAH1* gene and *PAH1*-knockout generation. **A**. Domain structure of the Pah1 protein, where the conserved N-lipin domain is shown in green. The asterix represents the conserved glycine residue, which, together with the aspartic acid residues in the HAD domain (orange), is necessary for the phosphatidic acid phosphatase activity of Pah1p. **B**. Knockout strategy used to delete the *PAH1* gene. Integration of the knockout cassette replaces the *PAH1* gene by the *LEU2* selection marker. The strategy for knocking out the *PAH1* gene was set up as described in Fickers *et al.* [11]. We generated a construct that includes the promoter and terminator fragment of the *PAH1* gene and a *LEU2* marker for selection. A knockout is obtained after double homologous recombination at the promoter and terminator sites of *PAH1*. **C**. After transformation with the *PAH1* knockout construct (P-*LEU2*-T), transformants were isolated and genotyped for the *PAH1* gene locus. Lane 1, 1 kb DNA marker (Promega). Lanes 2 and 3, PCR amplificate of the *PAH1* gene locus in, respectively, the wild type PO1d strain and an empty vector plasmid strain (expected wild type *PAH1* amplificate size is 3268 bp). Lane 4, PCR amplificate of the disrupted *PAH1* gene locus in a knockout strain (expected amplificate size is 2966 bp). Click here for file

Additional file 2: Figure S2 Adenosine A_2_A transgene mRNA expression in the wild type and *PAH1* deletion strains. Graph showing the normalized relative quantities of the adenosine A_2A_ receptor transgene mRNA in the wild type and *PAH1* deletion strains. A trend towards more transgene mRNA in the knockout strain can be observed, which however did not reach statistical significance (p = 0.083), even with 8 biological replicates. (Mann-Whitney U-test). Horizontal bars represent median with the interquartile ranges. Click here for file

Additional file 3: Table S1 qPCR primers of protein-coding genes. Click here for file

## References

[B1] Zoonens M Miroux B Expression of membrane proteins at the *Escherichia coli* membrane for structural studies Methods Mol Biol 2010 12 49 66 10.1007/978-1-60761-344-2_4 20099139

[B2] Rajakumari S Grillitsch K Daum G Synthesis and turnover of non-polar lipids in yeast Prog Lipid Res 2008 12 3 157 171 10.1016/j.plipres.2008.01.001 18258205

[B3] Pascual F Carman GM Phosphatidate phosphatase, a key regulator of lipid homeostasis Biochim Biophys Acta 2013 12 3 514 522 10.1016/j.bbalip.2012.08.006 22910056 PMC3549317

[B4] Santos-Rosa H Leung J Grimsey N Peak-Chew S Siniossoglou S The yeast lipin Smp2 couples phospholipid biosynthesis to nuclear membrane growth EMBO J 2005 12 1931 1941 10.1038/sj.emboj.7600672 15889145 PMC1142606

[B5] Nicaud JM Madzak C van den Broek P Gysler C Duboc P Niederberger P Gaillardin C Protein expression and secretion in the yeast *Yarrowia lipolytica* FEMS Yeast Res 2002 12 3 371 379 12702287 10.1016/S1567-1356(02)00082-X

[B6] Henry SA Kohlwein SD Carman GM Metabolism and regulation of glycerolipids in the yeast *Saccharomyces cerevisiae* Genetics 2012 12 317 349 10.1534/genetics.111.130286 22345606 PMC3276621

[B7] Graeve M Janssen D Improved separation and quantification of neutral and polar lipid classes by HPLC-ELSD using a monolithic silica phase: application to exceptional marine lipids J Chromatogr B Analyt Technol Biomed Life Sci 2009 12 1815 1819 10.1016/j.jchromb.2009.05.004 19493709

[B8] Guerfal M Ryckaert S Jacobs PP Ameloot P Van Craenenbroeck K Derycke R Callewaert N The *HAC1* gene from *Pichia pastoris*: characterization and effect of its overexpression on the production of secreted, surface displayed and membrane proteins Microb Cell Fact 2010 12 49 10.1186/1475-2859-9-49 20591165 PMC2905327

[B9] Tange Y Hirata A Niwa O An evolutionarily conserved fission yeast protein, Ned1, implicated in normal nuclear morphology and chromosome stability, interacts with Dis3, Pim1/RCC1 and an essential nucleoporin J Cell Sci 2002 12 22 4375 4385 10.1242/jcs.00135 12376568

[B10] Han GS Wu WI Carman GM The *Saccharomyces cerevisiae* Lipin homolog is a Mg2 + -dependent phosphatidate phosphatase enzyme J Biol Chem 2006 12 14 9210 9218 16467296 10.1074/jbc.M600425200 PMC1424669

[B11] Fickers P Le Dall MT Gaillardin C Thonart P Nicaud JM New disruption cassettes for rapid gene disruption and marker rescue in the yeast *Yarrowia lipolytica* J Microbiol Methods 2003 12 727 737 10.1016/j.mimet.2003.07.003 14607415

[B12] Fraser NJ Expression and functional purification of a glycosylation deficient version of the human adenosine 2a receptor for structural studies Protein Expr Purif 2006 12 129 137 10.1016/j.pep.2006.03.006 16630725

[B13] Sidrauski C Walter P The transmembrane kinase Ire1p is a site-specific endonuclease that initiates mRNA splicing in the unfolded protein response Cell 1997 12 1031 1039 10.1016/S0092-8674(00)80369-4 9323131

[B14] Oh MH Cheon SA Kang HA Kim JY Functional characterization of the unconventional splicing of *Yarrowia lipolytica HAC1* mRNA induced by unfolded protein response Yeast 2010 12 443 452 10.1002/yea.1762 20162530

[B15] Rasmussen SG Choi HJ Rosenbaum DM Kobilka TS Thian FS Edwards PC Burghammer M Ratnala VR Sanishvili R Fischetti RF Schertler GF Weis WI Kobilka BK Crystal structure of the human beta2 adrenergic G-protein-coupled receptor Nature 2007 12 383 387 10.1038/nature06325 17952055

[B16] Rasmussen SG DeVree BT Zou Y Kruse AC Chung KY Kobilka TS Thian FS Chae PS Pardon E Calinski D Mathiesen JM Shah ST Lyons JA Caffrey M Gellman SH Steyaert J Skiniotis G Weis WI Sunahara RK Kobilka BK Crystal structure of the beta2 adrenergic receptor-Gs protein complex Nature 2011 12 549 555 10.1038/nature10361 21772288 PMC3184188

[B17] Fakas S Qiu Y Dixon JL Han GS Ruggles KV Garbarino J Sturley SL Carman GM Phosphatidate phosphatase activity plays key role in protection against fatty acid-induced toxicity in yeast J Biol Chem 2011 12 29074 29085 10.1074/jbc.M111.258798 21708942 PMC3190715

[B18] Rockenfeller P Ring J Muschett V Beranek A Buettner S Carmona-Gutierrez D Eisenberg T Khoury C Rechberger G Kohlwein SD Kroemer G Madeo F Fatty acids trigger mitochondrion-dependent necrosis Cell Cycle 2010 12 14 2836 2842 10.4161/cc.9.14.12267 20647757

[B19] Blazeck J Reed B Garg R Gerstner R Pan A Agarwala V Alper HS Generalizing a hybrid synthetic promoter approach in *Yarrowia lipolytica* Appl Microbiol Biotechnol 2013 12 7 3037 3052 10.1007/s00253-012-4421-5 23053080

[B20] Blazeck J Liu L Redden H Alper H Tuning gene expression in *Yarrowia lipolytica* by a hybrid promoter approach Appl Environ Microbiol 2011 12 22 7905 7914 10.1128/AEM.05763-11 21926196 PMC3208987

[B21] Csaki LS Reue K Lipins: multifunctional lipid metabolism proteins Annu Rev Nutr 2010 12 257 272 10.1146/annurev.nutr.012809.104729 20645851 PMC3738581

[B22] Gaj T Gersbach CA Barbas CF 3rd ZFN, TALEN, and CRISPR/Cas-based methods for genome engineering Trends Biotechnol 2013 12 7 397 405 10.1016/j.tibtech.2013.04.004 23664777 PMC3694601

[B23] Echeverri CJ Perrimon N High-throughput RNAi screening in cultured cells: a user’s guide Nat Rev Genet 2006 12 5 373 384 16607398 10.1038/nrg1836

[B24] Hartner FS Ruth C Langenegger D Johnson SN Hyka P Lin-Cereghino GP Lin-Cereghino J Kovar K Cregg JM Glieder A Promoter library designed for fine-tuned gene expression in Pichia pastoris Nucleic Acids Res 2008 12 12 e76 10.1093/nar/gkn369 18539608 PMC2475614

[B25] De Pourcq K Vervecken W Dewerte I Valevska A Van Hecke A Callewaert N Engineering the yeast *Yarrowia lipolytica* for the production of therapeutic proteins homogeneously glycosylated with Man8GlcNAc2 and Man5GlcNAc2 Microb Cell Fact 2012 12 53 10.1186/1475-2859-11-53 22548968 PMC3512530

[B26] Barth G Gaillardin C Physiology and genetics of the dimorphic fungus *Yarrowia lipolytica* FEMS Microbiol Rev 1997 12 219 237 10.1111/j.1574-6976.1997.tb00299.x 9167256

[B27] Baharaeen S Vishniac HS A fixation method for visualization of yeast ultrastructure in the electron microscope Mycopathologia 1982 12 19 22 10.1007/BF00588651 6803162

[B28] Hellemans J Mortier G De Paepe A Speleman F Vandesompele J qBase relative quantification framework and software for management and automated analysis of real-time quantitative PCR data Genome Biol 2007 12 R19 10.1186/gb-2007-8-2-r19 17291332 PMC1852402

[B29] Folch J Lees M Sloane Stanley GH A simple method for the isolation and purification of total lipids from animal tissues J Biol Chem 1957 12 497 509 13428781

