# Supplementary figures and images for: Enhanced membrane protein expression by engineering increased intracellular membrane production
Source: Microb Cell Fact. 2013 Dec 9;12:122. doi: 10.1186/1475-2859-12-122 (PMC3878919; doi:10.1186/1475-2859-12-122)

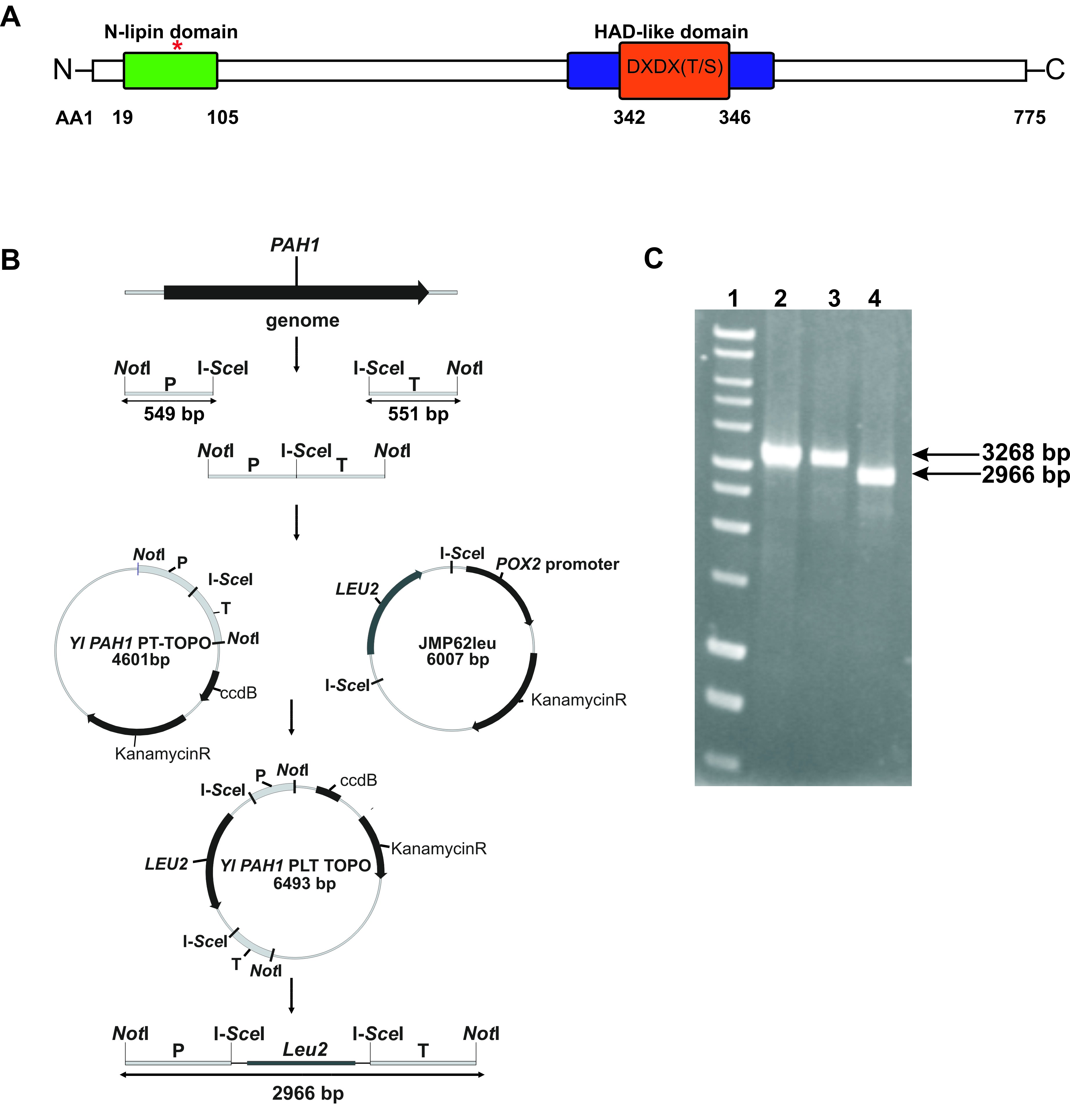

Supplement: Additional file 1: Figure S1 — Yarrowia lipolytica PAH1 gene and PAH1-knockout generation. A. Domain structure of the Pah1 protein, where the conserved N-lipin domain is shown in green. The asterix represents the conserved glycine residue, which, together with the aspartic acid residues in the HAD domain (orange), is necessary for the phosphatidic acid phosphatase activity of Pah1p. B. Knockout strategy used to delete the PAH1 gene. Integration of the knockout cassette replaces the PAH1 gene by the LEU2 selection marker. The strategy for knocking out the PAH1 gene was set up as described in Fickers et al. [11]. We generated a construct that includes the promoter and terminator fragment of the PAH1 gene and a LEU2 marker for selection. A knockout is obtained after double homologous recombination at the promoter and terminator sites of PAH1. C. After transformation with the PAH1 knockout construct (P-LEU2-T), transformants were isolated and genotyped for the PAH1 gene locus. Lane 1, 1 kb DNA marker (Promega). Lanes 2 and 3, PCR amplificate of the PAH1 gene locus in, respectively, the wild type PO1d strain and an empty vector plasmid strain (expected wild type PAH1 amplificate size is 3268 bp). Lane 4, PCR amplificate of the disrupted PAH1 gene locus in a knockout strain (expected amplificate size is 2966 bp). [file 1475-2859-12-122-S1.jpeg]

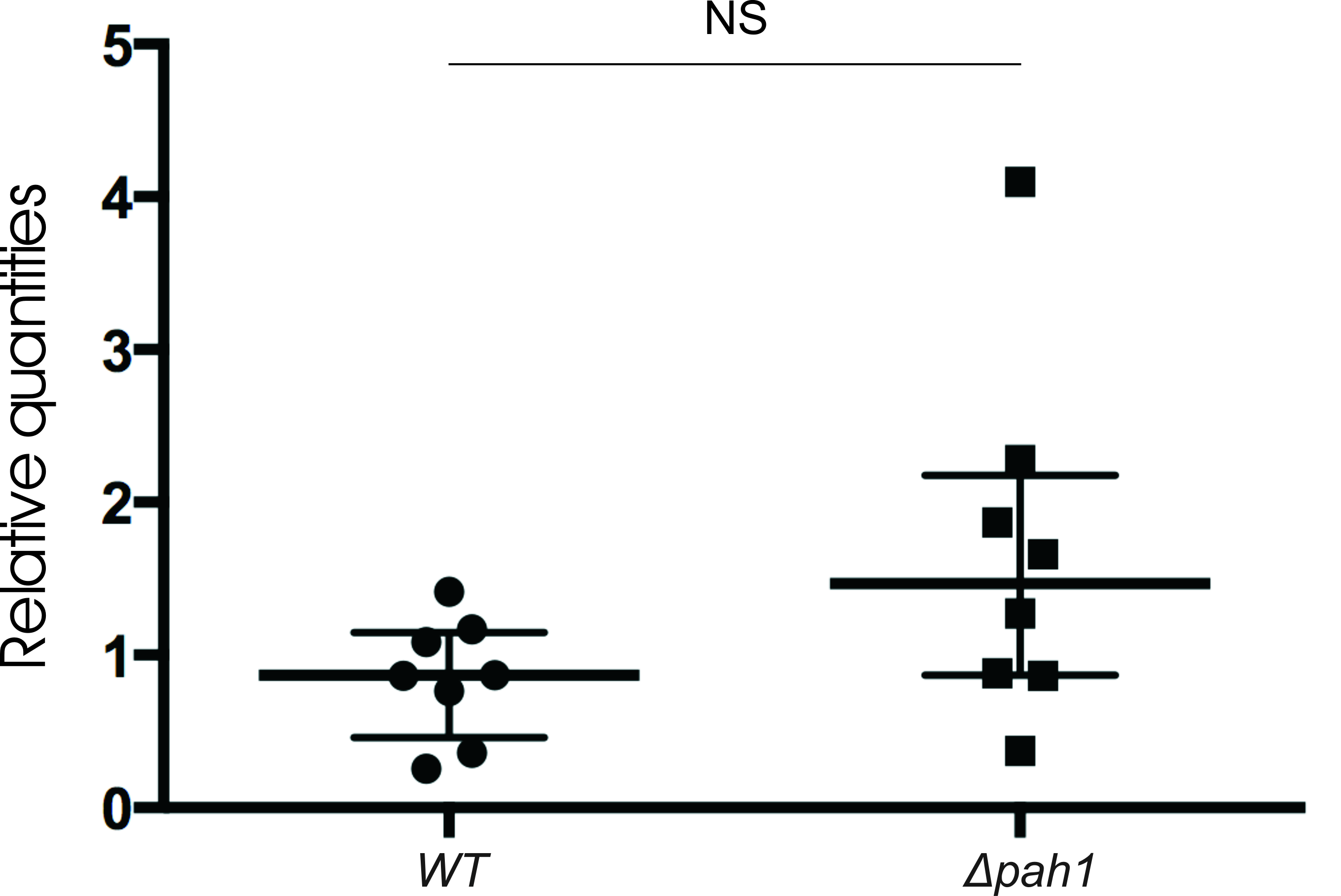

Supplement: Additional file 2: Figure S2 — Adenosine A2A transgene mRNA expression in the wild type and PAH1 deletion strains. Graph showing the normalized relative quantities of the adenosine A2A receptor transgene mRNA in the wild type and PAH1 deletion strains. A trend towards more transgene mRNA in the knockout strain can be observed, which however did not reach statistical significance (p = 0.083), even with 8 biological replicates. (Mann-Whitney U-test). Horizontal bars represent median with the interquartile ranges. [file 1475-2859-12-122-S2.tiff]
