# Supplementary material for: Enhanced membrane protein expression by engineering increased intracellular membrane production
Source: Microb Cell Fact. 2013 Dec 9;12:122. doi: 10.1186/1475-2859-12-122 (PMC3878919; doi:10.1186/1475-2859-12-122)
Supplement: Additional file 3: Table S1 — qPCR primers of protein-coding genes. [file 1475-2859-12-122-S3.doc]

**Supplementary Table 1.** qPCR primers of protein-coding genes

| Reference/Target gene | Sequence |
| --- | --- |
| PGK Fw | 5’-CTCATCGACA ACCTACTTG-3’ |
| PGK Rev | 5’-TTTCCAATCT TGACGTTCTC-3’ |
| QCR9 Fw | 5’-TTTGTCTTTG ACATGACCTT-3’ |
| QCR9 Rev | 5’-ATATCCTTCC ATTGTTTGCC-3’ |
| A2A Fw | 5’-CATGGTGTAC TTCAACTTCT-3’ |
| A2A Rev | 5’-GAAGATCCGC AAATAGACAC-3’ |
